# EFFECT OF A HEARING INTERVENTION COMPARED TO EDUCATION CONTROL ON SELF-REPORTED PHYSICAL ACTIVITY: ACHIEVE STUDY

**DOI:** 10.1093/geroni/igae098.0611

**Published:** 2024-12-31

**Authors:** Pablo Martinez Amezcua

**Affiliations:** Johns Hopkins University, Baltimore, Maryland, United States

## Abstract

Hearing loss is associated with lower physical activity among older adults. However, whether the treatment of hearing loss influences engagement in physical activity remains unknown. Using data from the Aging Cognitive Health Evaluation in Elders (ACHIEVE) study, a 1:1 randomized control trial of a hearing care intervention (including the provision of hearing aids) compared to a health education control group, we investigated the effect hearing care vs. control on self-reported participation in any moderate-to-vigorous physical activity (MVPA) and frequently walking during leisure time (defined as reporting leisurely walking often or very often) among community-dwelling older adults. Among 977 participants (age 70-84; female: 53.5%; White: 87.8%) with hearing loss recruited from the Atherosclerosis Risk in Communities Study (ARIC) (n=238) and de novo from the community (n=739), over three years there was a 3% reduction in the proportion of participants who engaged in MVPA (57.5% to 54.7%), but a 5% increase in leisurely walking (29.2% to 34.4%). Participants in the hearing intervention had 5% and 7% higher odds of reporting MVPA and leisurely walking over three years than those in the control group (OR MVPA=1.05, 95% CI: 0.96, 1.14; OR walking=1.07, 95%: 0.96, 1.19). These associations were stronger, 9% and 41%, among de novo than ARIC participants (de novo: OR MVPA=1.09, 95% CI: 0.79, 1.53; OR walking=1.41, 95% CI: 0.97, 2.05). Findings suggest addressing hearing loss may increase physical activity, mainly leisurely walking. Further research is warranted to explore additional factors influencing physical activity in older adults with hearing loss.

